# Association of Imposter Syndrome and psychological well-being in the doctoral process - how they are influenced by experiences of discrimination, social support and belonging

**DOI:** 10.1192/j.eurpsy.2025.1862

**Published:** 2025-08-26

**Authors:** J. Pokrandt, P. J. Buspavanich, M. Berger, G. Stadler, J. B. Smith

**Affiliations:** 1 Charité Universitätsmedizin Berlin; 2 Brandenburg Medical School Theodor Fontane, Berlin, Germany

## Abstract

**Introduction:**

Individuals who develop an Imposter Syndrome do not attribute objective successes to their own abilities and competences, but rather believe that they are not intelligent enough, and are sometimes convinced that they have deceived others. It is known that the Imposter Syndromehas various effects on health. In particular, it affects the mental health of the individuals affected. Although it is known that the manifestation of the Imposter Syndrome is higher in marginalized groups in academic fields. Whether the intersectionality of the relevant diversity domains and personal resources such as social support and belonging have an influence on the extent of the imposter phenomenon has not yet been investigated.

**Objectives:**

The aim of the study was to determine the association between imposter syndrome to the psychological well-being of supervisors and doctoral students in the doctoral process considering the mediating influence of experiences of discrimination, social support and belonging.

**Methods:**

A six-month program was developed to accompany the promotion process. A total of seven groups were conducted from April 2024 to May 2025. At the beginning of the programme, baseline data was collected using The WHO-5 Well Being Questionnaire, the Sense of Belonging Questionnaire, the F-Sozu-6 Questionnaire, the Diversity minimal item set and the Clance Imposter Phenomenon Scale.

**Results:**

Preliminary data show that the Imposter syndrome is widespread among supervisors and doctoral students. Individuals who perceive themselves as belonging to multiple diversity domains tend to exhibit diminished psychological well-being, particularly when considering the intersectionality of these domains.

**Conclusions:**

The findings of this study indicate that the Imposter syndrome should be addressed in an accompanying doctoral program with a focus on gender- and diversity aspects. Diversity domains, social support and sense of belonging should be considered more frequently in the development of academic career interventions.

**Disclosure of Interest:**

None Declared

